# Optimizing Combinations of Flavonoids Deriving from Astragali Radix in Activating the Regulatory Element of Erythropoietin by a Feedback System Control Scheme

**DOI:** 10.1155/2013/541436

**Published:** 2013-04-30

**Authors:** Hui Yu, Wendy L. Zhang, Xianting Ding, Ken Y. Z. Zheng, Chih-Ming Ho, Karl W. K. Tsim, Yi-Kuen Lee

**Affiliations:** ^1^State Key Laboratory of Analytical Chemistry for Life Science, Nanjing University, Nanjing, Jiangsu, China; ^2^Department of Mechanical Engineering, The Hong Kong University of Science and Technology, Clear Water Bay Road, Hong Kong; ^3^Division of Life Science and Center for Chinese Medicine, The Hong Kong University of Science and Technology, Clear Water Bay Road, Hong Kong; ^4^School of Biomedical Engineering, Med-X Research Institute, Shanghai Jiao Tong University, Shanghai, China; ^5^Center for Cell Control, Mechanical and Aerospace Engineering Department, Biomedical Engineering Department, University of California, Los Angeles, CA 90095-1597, USA

## Abstract

Identifying potent drug combination from a herbal mixture is usually quite challenging, due to a large number of possible trials. Using an engineering approach of the feedback system control (FSC) scheme, we identified the potential best combinations of four flavonoids, including formononetin, ononin, calycosin, and calycosin-7-O-**β**-D-glucoside deriving from Astragali Radix (AR; Huangqi), which provided the best biological action at minimal doses. Out of more than one thousand possible combinations, only tens of trials were required to optimize the flavonoid combinations that stimulated a maximal transcriptional activity of hypoxia response element (HRE), a critical regulator for erythropoietin (EPO) transcription, in cultured human embryonic kidney fibroblast (HEK293T). By using FSC scheme, 90% of the work and time can be saved, and the optimized flavonoid combinations increased the HRE mediated transcriptional activity by ~3-fold as compared with individual flavonoid, while the amount of flavonoids was reduced by ~10-fold. Our study suggests that the optimized combination of flavonoids may have strong effect in activating the regulatory element of erythropoietin at very low dosage, which may be used as new source of natural hematopoietic agent. The present work also indicates that the FSC scheme is able to serve as an efficient and model-free approach to optimize the drug combination of different ingredients within a herbal decoction.

## 1. Introduction

 Traditional Chinese medicine (TCM) has played an important role in primary health care in China of over thousands of years [1]. In contrast to isolated, bioactive, single natural products in Western medicine, TCM uses a mixture of active ingredients; this represents a holistic approach in disease prevention. TCM has attracted a lot of attention for serving as complementary health food supplements with low toxicity and fewer complications [2, 3]. Astragali Radix (AR; Huangqi), the dried root of *Astragalus membranaceus* (Fisch.) Bunge or *A. membranaceus* (Fisch.) Bunge var. *mongholicus* (Bunge) P. K. Hsiao, is one of the most widely used Chinese herbs as a health food supplement to reinforce “Qi” (vital energy) [4]. Pharmacological study has demonstrated that the water extract of AR possesses many biological functions, including hepatoprotective effects, neuroprotective effects against ischemic brain injury, hematopoietic, antioxidative, antihypertensive, immunological properties, cardiotonic, and antiaging activities [5, 6].

Previous study also showed that the AR extract could improve hematopoietic functions by regulating erythropoietin (EPO) expression. EPO is an erythrocyte-specific hematopoietic growth factor produced by kidney and liver [7]. Failure to increase the amount of circulating EPO under hypoxia stress can lead to anemia [8]. Hypoxia response element (HRE), a critical regulator for EPO transcription, is located on the promoter region of the EPO gene. Under hypoxia condition, the activated hypoxia-induced factor (HIF) binds onto HRE and subsequently initiates EPO gene expression [9]. The AR regulating EPO expression was through an induction of the transcriptional activity of HRE [10]. One of major components in AR was flavonoid, for example, formononetin, ononin, calycosin, and calycosin-7-O-**β**-D-glucoside. These four flavonoids can induce the expression of EPO [11]; however, the effect of a combination of these flavonoids has not been revealed. Indeed, the combined mixture is mimicking partly the scenario of a herbal mixture.

A major problem in combinatorial therapies lies in the number of possible combinations [12, 13], which becomes more challenging in optimizing Chinese herbal mixtures. Besides, the combined effect of numerous constituents within a herbal composite prescription is hard to validate [14]. Previous study indicated that different experimental methods have been used in discovering combinatorial therapies. Li et al. established an algorithm termed NIMS (Network Target-Based Identification of Multicomponent Synergy) to prioritize synergistic agent combinations in a high throughput way [15]. Both the topology score and agent score were proposed for the evaluation of agent interactions; Yan et al. developed a systematic simplification framework for drug combination design by combining simulation and system reaction network topology analysis [16]. Among different classes of strategy, Wong et al. developed a feedback system control (FSC) scheme to implement an iterative stochastic search [17]. FSC efficiently discovered potent combinations for inhibiting virus infection of fibroblasts, in only tens of iterations out of one hundred thousand possible trials. Recently, Tsutsui et al. further extended the FSC for parallel searching and identified a unique combination of three combined inhibitors that enables the maintenance of human embryonic stem cells [18]. Here, we aimed to optimize a herbal mixture for therapeutic goals by using the FSC scheme, and therefore the optimization of AR-derived flavonoid combinations was evaluated as an initial example. Having the FSC, we were able to quickly pinpoint the optimal flavonoid combinations for maximizing the HRE-mediated transcriptional activity.

## 2. Materials and Methods

### 2.1. Plant Materials and Chemicals

Three-year-old AR, the dry roots of *A. membranaceus* var. *mongholicus*, was collected from Shanxi province [4]. The authentication of plant materials was performed morphologically by Dr. Tina Dong of The Hong Kong University of Science and Technology (HKUST) during the field collection. The corresponding voucher no. 02-10-4, as forms of the whole plant were deposited in Center for Chinese Medicine, HKUST. Formononetin, calycosin, ononin, and calycosin-7-O-**β**-D-glucoside were purchased from Weikeqi Biotechnology Co. (Sichuan, China). The purities of these marker chemicals, confirmed by high-performance liquid chromatography (HPLC), were higher than 98.0%. Analytical- and HPLC-grade reagents were from Merck (Darmstadt, Germany).

### 2.2. Preparation of AR Extracts and Chemicals

Dry roots of AR (50 g) were extracted twice with distilled water (400 mL) at 100°C for 2 hours. The extract was centrifuged at 3,000 g for 10 min. The supernatant was freeze-dried (yield = 14.56 g) and kept at −20°C. For standardization of AR extract, an Agilent 1200 series system (Agilent, Waldbronn, Germany), equipped with a degasser, a binary pump, an autosampler, and a thermostated column compartment was used for the analysis. Chromatographic separations were carried out on an Agilent Eclipse XDB-C18 column (4.6 mm × 250 mm, 5 *μ*m) with 0.1% formic acid (as Solvent A) and acetonitrile (as Solvent B) at a flow rate of 1.0 mL/min at room temperature. A linear gradient elution was applied from 15%–20% B at 0–10 min, 20% B at 10–20 min, 20%–34% B at 20–45 min, 34%–48% B at 45–55 min, 48%–65% B at 55–70 min, and 65%–80% B at 70–80 min, and the equilibration time of gradient elution was 10 min. Ten *μ*L of the samples (after filtration with a 0.45 *μ*m Millipore filter) were injected, and signals were detected at 280 nm with UV detection. A standardized AR extract by calibrating different chemical markers was established. For biological analysis, the dried standardized extract was dissolved in phosphate-buffered saline and filtered through a 0.22 *μ*m filter before use. The pure flavonoids were dissolved by dimethyl sulfoxide before use.

### 2.3. DNA Transfection in Culture

Human embryonic kidney (HEK) 293T fibroblast cell is an excellent *in vitro* model in studying the physiological regulation of EPO expression, which is sensitive to hypoxia stress [7]. HEK 293T cell line was obtained from the American Type Culture Collection (ATCC, Manassas, VA) and cultured according to previous reports [11]. The HRE (5′-TCG AGG CCC TAC GTG CTG TCT CAC ACA GCC TGT CTG ACG-3′) derived from human EPO gene contains a highly conserved HIF-1 binding site (5′-TAC GTG-3′) and other unique cis-acting sequences (5′-CAC AG-3′) that are functionally essential for hypoxic induction [11, 19]. Six HREs were synthesized, concatemerized, and then cloned in tandem (head-to-tail orientation) into pBI-GL vectors (BD Biosciences Clontech, San Jose, CA) that had a downstream reporter of firefly luciferase gene [11]. This vector was named as pHRE-Luc [19]. Cultured HEK293T cells were transiently transfected with pHRE-Luc by the calcium phosphate precipitation method [20]. The transfection efficiency was over 80%, as determined by another control plasmid of having a *β*-galactosidase cDNA, under a cytomegalovirus enhancer promoter. The treatment of flavonoids, or AR extract, was done on transfected HEK293T cells. After 2 days, the cell lysates were collected for luciferase assay. In order to validate the response of the pHRE-Luc in transfected HEK293T cells, the cultures were exposed to hypoxia, serving as a positive control. The authentication of pHRE-Luc was confirmed by its activation in exposing to mineral oil layering and application of CoCl_2_ at 50 mM, and both methods were frequently used to mimic the effect of hypoxia [11]. 

### 2.4. Luciferase and Other Assays

The luciferase assay was performed by a commercial kit (Tropix Inc., Bedford, MA). In brief, cultures were lysed by a buffer containing 100 mM potassium phosphate buffer (pH 7.8), 0.2% TritonX-100, and 1 mM dithiothreitol. The cell lysate was centrifuged in 13,200 rpm (16,000 ×g) for 5 min in 4°C, and 50 *μ*L of the supernatant was transferred to the assay plate and set on the luminance reading machine (FLUOstar OPTIMA, BMG, Germany). The readings of luminance intensity were equalized by the protein concentration of lysates, and the data indicated to the luciferase activities of the samples. Protein concentrations were measured routinely by Bradford's method with a kit from Bio-Rad Laboratories (Hercules, CA).

## 3. Results

### 3.1. AR and Flavonoids Induce HRE-Mediated Transcriptional Activity

**Figure 1:**
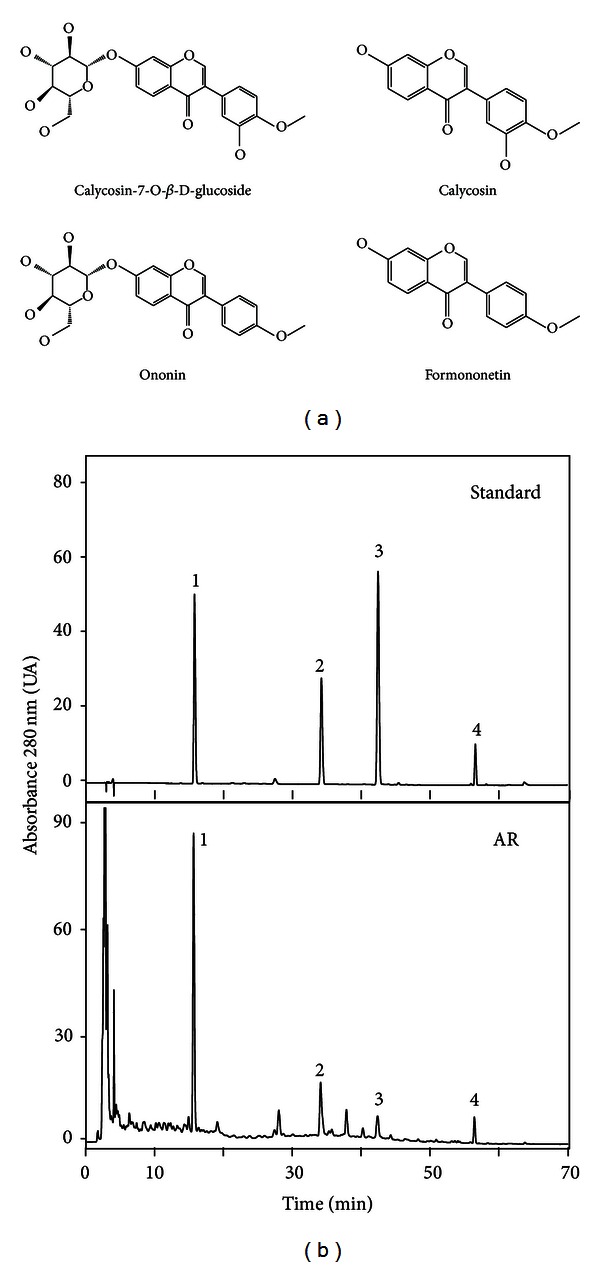
Chemical standardization of AR extract by HPLC fingerprint analysis. (a) Calycosin, calycosin-7-O-*β*-D-glucoside, formononetin, and ononin are the major flavonoids contained within AR water extract. (b) In a HPLC fingerprint at an absorbance of 280 nm, the peaks corresponding to calycosin-7-O-*β*-D-glucoside (1), ononin (2), calycosin (3), and formononetin (4) were identified as standards (upper panel). The chemical amounts of these four compounds contained within the water extract of AR were calculated according to the HPLC results (lower panel). Representative chromatograms are shown; *n* = 3.

**Figure 2:**
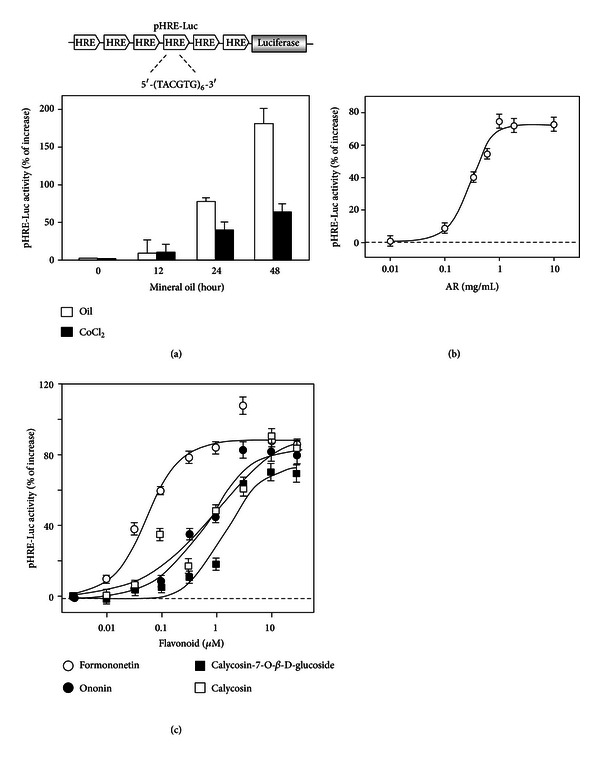
The AR extract and flavonoids stimulated the HRE-mediated transcriptional activity in cultured HEK293T cells. (a) Six repeats of hypoxia responsive elements (HRE: 5′-TCG AGG CCC TAC GTG CTG TCT CAC ACA GCC TGT CTG ACG-3′) were subcloned in an expression vector of luciferase named as pHRE-Luc (upper panel). Cultured HEK293T cells, transfected with pHRE- Luc, were treated with CoCl_2_ (50 mM) or mineral oil layering for 0 to 48 hours. The cell lysates were subjected to luciferase assay to measure the transcriptional activity driven by HRE (lower panel). (b and c) The pHRE-Luc-transfected HEK293T cells were treated with AR extracts (b) and flavonoids (c) for 48 hours to determine the promoter-driven luciferase (pHRE-Luc) activity. Values are expressed as the percentage of increase to basal reading (untreated culture) and in mean ± SD, where *n* = 4, each with triplicate samples.

Calycosin, calycosin-7-O-*β*-D-glucoside, formononetin, and ononin are the major flavonoids contained within AR water extract (Figure 1(a)), which have inductive effect in EPO expression [10, 11]. HPLC analysis indicated that a standardized AR extract should contain the following marker compounds (*μ*g/1 g; mean ± SD, *n* = 4) (Figure 1(b)): calycosin (212.69 ± 21.1), calycosin-7-O*-*β**-D-glucoside (238.4 ± 19.5), formononetin (150.12 ± 13.7), and ononin (85.66 ± 8.4). We first examined the abilities of AR extract and the flavonoids in the stimulation of HRE mediated transcriptional activity. The AR extract and four flavonoids, formononetin, ononin, calycosin, and calycosin-7-O-**β**-D-glucoside, were applied onto pHRE-Luc-transfected fibroblasts for two days. The authentication of pHRE-Luc was confirmed by its activation in exposing to mineral oil layering or CoCl_2_ treatment, which was frequently used to mimic the effect of hypoxia [10]. Under the hypoxia by oil layering or CoCl_2_, the expression of pHRE-Luc was robustly induced in a time-dependent manner (Figure 2(a)). The AR extract and flavonoids showed significant induction on the pHRE-Luc activity in a dose-dependent manner (Figures 2(b) and 2(c)). The maximal induction by AR extract was over 70% of increase as compared to the background. Formononetin was the most potent flavonoid in the HRE activation, which induced a maximum over 90% at 1 *μ*M, and the EC_50_ value was ~0.05 *μ*M. The EC_50_ of ononin, calycosin, and calycosin-7-O-**β**-D-glucoside were 0.56, 0.66, and 1.47 *μ*M, and maximal inductions were 80%, 83%, and 70% of increase, respectively (Figure 2(c)).

### 3.2. Optimization Strategy with Feedback System Control (FSC) Scheme

**Figure 3:**
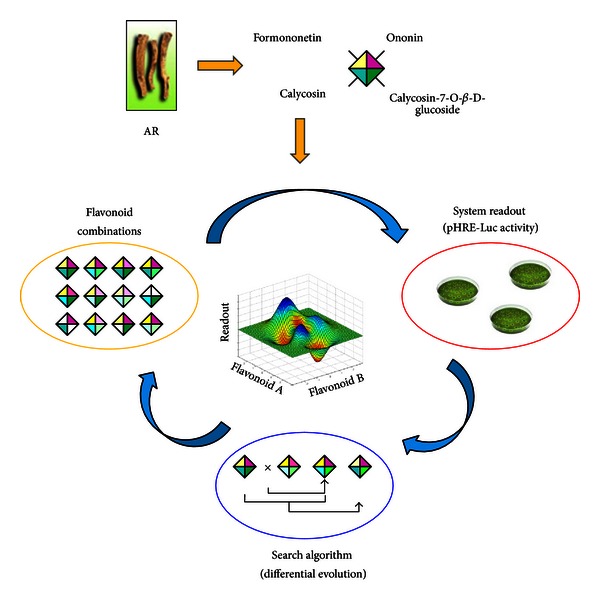
Optimization of the flavonoid combination by feedback system control (FSC) scheme. Feedback system control (FSC) scheme was used to optimize the flavonoid combinations. As trial combinations, the drug combinations of four flavonoids were applied onto pHRE-Luc-transfected fibroblasts. The HRE-mediated transcriptional activity was used as the system readout to calculate the fitness of combinations. Differential evolution (DE) algorithm linked the drug combinations and the system readout as to generate new trial combinations in the next iteration.

The FSC scheme consists of an iterative closed-loop of three operations, including formation of drug combinations, experimental readouts, and search algorithm (Figure 3). As the trials, a group of drug combinations selected from the parametric search space were applied in cultured cells. Induced cellular activities served as the fitness in drug effect evaluation. Based on the cellular responses, the search algorithm linked the cellular readouts and the drug combinations and therefore generated new combinations for subsequent iteration of experimental tests.

 The four flavonoids were mixed and dissolved in dimethyl sulfoxide as the trial combinations. Based on the dose response of individual flavonoids (as from Figure 2(c)), we determined six concentration levels (0, 0.016, 0.08, 0.4, 2, and 10 *μ*M) of each flavonoid to fully cover the effective range. Flavonoid combinations composed a parametric search space of 1,296 possible trials. The number of possible combinations rapidly increased with the number of flavonoids and concentration levels, as in an exponential form. Six index numbers of 0, 1, 2, 3, 4, 5 were assigned for the concentration levels of 0, 0.016, 0.008, 0.4, 2, 10 *μ*M, respectively; these index numbers were used later in the differential evolution search algorithm. 

 The success of FSC scheme heavily relied on the cellular readouts that closely mimicked the desired biological activity and the proper controls to evaluate the effects of the drug combinations. Our goal was to search for potent drug combinations that could stimulate EPO expression, and thus we used the HRE-mediated transcriptional activity as the initial readout. Since HRE is located on the promoter of EPO gene, the induced HRE activity can subsequently trigger the transcription of EPO gene [19].

 The search algorithm plays an important role in the FSC scheme, which determines the efficiency and accuracy of the exploration. We used the differential evolution algorithm to perform a parallel exploration in the chemical optimization, due to its easy operation and previous successful application [16, 21, 22]. The principle and detailed application of the differential evolution algorithm in our FSC scheme were introduced in the supporting information (Figure 1(S), Supporting information, see supplementary materials available online at http://dx.doi.org/10.1155/2013/541436). Parameters, including the number of population (NP) and crossover probability (CR), were modified to better fit our system.

### 3.3. Optimization of Flavonoid Combination Using FSC

**Figure 4:**
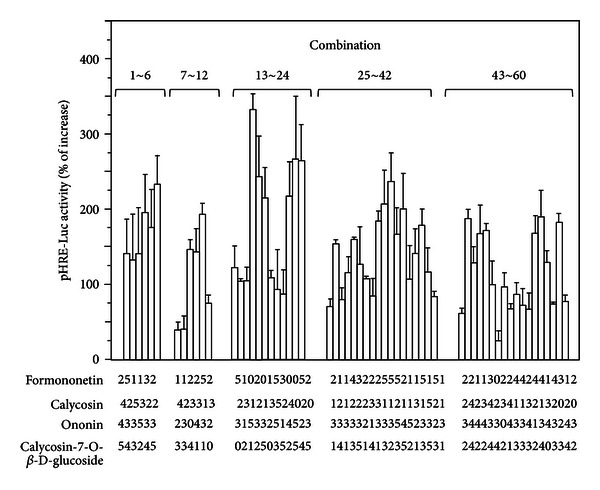
The HRE-mediated transcriptional activity induced by the 60 trial combinations in five iterations. The pHRE-Luc-transfected HEK293T cells were treated by the 60 trial combinations in five iterations, that is, 1–6, 7–12, 13–24, 25–42, and 43–60. The integer index of four flavonoids represented the corresponding concentration in each trial shown under each combination, that is, the index numbers of 0, 1, 2, 3, 4, 5 are corresponding to the final concentrations of 0, 0.016, 0.008, 0.4, 2, 10 *μ*M. After 48 hours, the promoter-driven luciferase (pHRE-Luc) activity was determined. The response to mineral oil layering, a positive control, was as effective as in Figure 2(a). Values are expressed as the percentage of increase to basal reading (untreated culture) and in mean ± SD, where *n* = 4, each with triplicate samples.

We iteratively evaluated 60 trial combinations in five iterations, each with at least triplicated samples (Figure 4). To determine the collaborative role of four flavonoids on pHRE-Luc-transfected HEK 293T cells, we randomly selected six different combinations of the four flavonoids as trial combinations in the first iteration, using a random number generator in MATLAB (MathWorks). The HRE-mediated transcriptional activities, induced by the combinations, were found to be higher than 130%. The highest response obtained in the first iteration was 232% increase of the control, induced by the 6th combination (0.08, 0.08, 0.4, 10 *μ*M for formononetin, ononin, calycosin and calycosin-7-O-**β**-D-glucoside, resp.). Comparing with AR extract and individual flavonoid, higher activities induced by the drug combinations suggested possible collaborative effects among the flavonoids.

**Figure 5:**
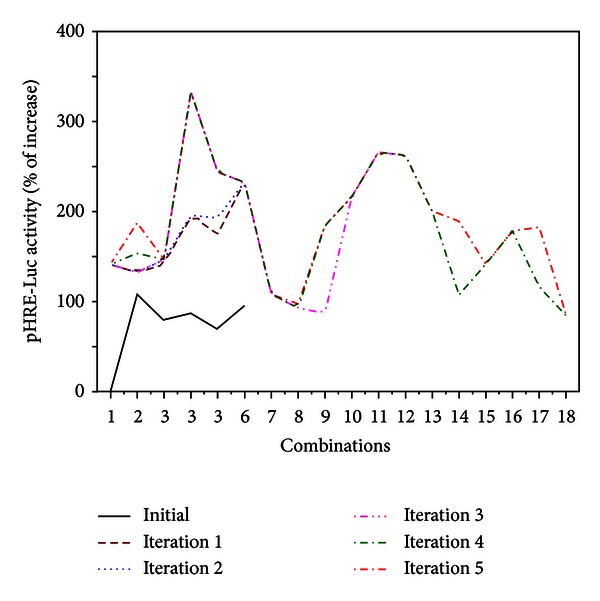
The HRE-mediated transcriptional activity induced by the winning population of flavonoid combinations in each iteration In each iteration, the *x*-axis represents the *i*th combination in current winning population. Winning combinations are determined after competition between original combinations and trial combinations, as in Figure 1(S). Values are expressed as the mean value in Figure 4. Initial combinations are comprised of control, AR extract, and individual flavonoid. The FSC scheme iteratively drove combinations in the population towards higher HRE-mediated transcriptional activity; that is, the best response of 3rd combination in the population was found in iteration 3, and carried over through iterations 4 and 5.

 In the following iterations, we then attempted to optimize the combinations of the four flavonoids. The trial combinations were generated by the differential evolution (DE) algorithm, also using the MATLAB. To avoid being trapped in local maximum responses, the number of population was increased from 6 to 12, and finally 18 after iteration 3, and crossover probability was changed from 0.5 to 0.9 after iteration 2. The FSC scheme iteratively updated the potent drug combinations towards better system performance, after the competition between original combinations and trial combinations in each iteration. (Figure 5). After the third iteration, by which we accumulatively tested 24 trials out of 1,296 possible combinations, we identified the 16th combination, that is, 0.08, 0.08, 0.4, 0.08 *μ*M of the four flavonoids. This combination of flavonoids showed a ~3-fold improvement (333%) in stimulating HRE-mediated transcriptional activity compared with individual flavonoids (see Figure 4). This unique combination was carried over through iterations 4 and 5, showing promising drug potency.

 Distinct effects of drug combinations in stimulating HRE-mediated transcriptional activity indicated the complicated reciprocity among flavonoids. Out of the 60 trials, we observed that 13 trial combinations induced lower HRE activation than AR and individual flavonoid. The minimum response was observed to be 39% at the 49th combination, that is, 0.016, 2, 0.08, 0.4 *μ*M, which was ~2-fold decrease as compared with individual flavonoid (see Figure 4). Meanwhile, 16 potent combinations induced higher HRE-mediated transcriptional activity than 180% increase of control. Typical dosages of formononetin, ononin, calycosin and calycosin-7-O-**β**-D-glucoside in 1 mg/mL of AR extract were around 0.56 *μ*M, 0.16 *μ*M, 0.69 *μ*M, and 0.513 *μ*M, respectively [23–25]. Among the 16 potent combinations, the 16th (0.08, 0.08, 0.4, 0.08 *μ*M) and 18th combinations (0.016, 0.4, 0.08, 0 *μ*M) reduced the required dosage by ~10-fold compared with the amounts within AR water extract (see Figure 4).

**Figure 6:**
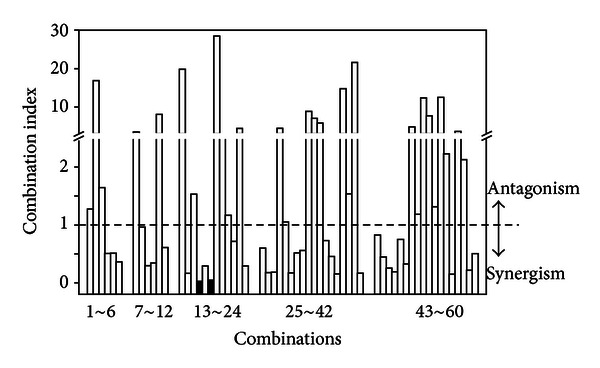
Combination index of the 60 trial combinations. The combination indexes (CIs) < 1, = 1, and > 1 indicate synergism, additive effect, and antagonism, respectively. The calculation of CI was accord with the theory of Chou. Sixty combinations out of 5 five iterations are shown here. The 16th and 18th combinations (black bars) showed the strongest synergism, that is, the smallest CI value.

The AR flavonoids possess a lot of biological functions as described previously [26]; however, the usage of combined flavonoids in a collaborative way is still challenging. To reveal if the flavonoids can work in a synergistic or antagonistic way in activating HRE mediated transcriptional activity, here we used the well-known median-effect equation proposed by Chou [27–30] (Figure 2(S), Supporting information). In Chou's theory, the combination index (CI) was used as the evaluation of synergistic effect. CI < 1, CI = 1, and CI > 1 indicate synergism, additive effect, and antagonism, respectively. The CI values of the 60 trial combinations in our FSC optimization were quite different from each other, ranging from 0.033 to 28.5, showing that the synergistic and antagonistic effects were closely related with the mixing ratio of flavonoids within the drug combinations (Figure 6). Interestingly, the minimum CI values were observed also in the 16th and 18th combinations, to be 0.033 and 0.044, respectively. According to Chou's summary [30], the CI value smaller than 0.1 indicated possible very strong synergism among flavonoids to stimulate the HRE-mediated transcriptional activity, although dose-dependent studies of the combinations were required to obtain further information.

There is no guarantee that the combination we identified would be the best out of the 1,296 possible combinations, until we have exhaustively tested all the combinations. However, we can testify the effectiveness of this FSC scheme by simulation. Four different benchmark functions were used to mimic the biological system readout. The differential evolution algorithm was implemented exactly the same as used in our flavonoid optimization. Simulation results suggested that the FSC scheme was able to quickly pinpoint the optimal solution (Figure 3(S), Supporting information). Thus, our FSC optimization could be regarded as the stochastic optimal flavonoid combinations of the 1,296 possible combinations.

## 4. Discussion

 The role of flavonoids in regulating the EPO expression has been known [10]. However, the detailed synergistic or antagonistic interaction among flavonoids has not been revealed yet, which therefore stimulates the engineering approach to optimize the flavonoid combinations by FSC scheme. By having FSC approach, we have saved over 90% of the laborious and time-consuming work that is required in exhaustively testing of all possible combinations. Two unique combinations, (0.08, 0.08, 0.4, 0.08) *μ*M and (0.016, 0.4, 0.08, 0) *μ*M of formononetin, ononin, calycosin, and calycosin-7-O-**β**-D-glucoside, were identified to be highly effective in stimulating HRE-mediated transcriptional activity, which was increased ~3-fold by the two optimized combinations, while the AR herbal extract and individual flavonoid can only induce the transcriptional activity by about 80%. The concentration of these four flavonoids that we used here is much lower than that in the herbal extract of AR. Not only in cultured cells, our preliminary results indicated that the application of optimized combination of flavonoids could achieve maximal activation of red blood cell production in animal studies (Zhang et al., unpublished result). By using the FSC scheme as described here, the transcriptional activity of EPO was greatly increased, and the amount of flavonoids used was reduced by ~10-fold.

The successful application of FSC scheme in optimizing the combination of four flavonoids provides hints in study of TCM formulae. According to TCM theory, the herbal formulae should be prepared in a unique methodology having specific combination of different herbs as a formula (named as *Fu Fang*). In general, the combination amongst different herbs within a decoction will directly affect the pharmacological properties of a herbal formula. Indeed, our previous work has supported the usage of the best combination of two herbs in Danggui Buxue Tang, a traditional herbal decoction. Having the best combination of herbs, this herbal decoction possesses enriched chemical and biological properties [23]. However, the major problem in the optimization of herbal mixtures is the number of possible combinations. For example, over thousands of possible herb combinations could be found even in a herbal decoction having two herbs. Experimentally, it is impossible to try all these combinations of herbal extracts in optimizing a TCM formula. Having FSC scheme, only about tens of trials are required to optimize the combination, and almost 90% of the work can be trimmed. Thus, the FSC scheme as described here can be used as a new approach to optimize the combination of herbal extracts in TCM formula, which will provide more evidence for the better use of herbal mixture. Moreover, the FSC scheme may be further developed to predict the drug combinations [31].

## 5. Conclusions 

In summary, it can be stated that optimized combinations of flavonoids have a strong HRE-mediated transcriptional activity, which suggests that the optimized flavonoids could have good activity in stimulating the regulatory element of erythropoietin at a very low concentration. The application of FSC scheme could be able to identify potent drug combination of ingredients within herbal medicines, as well as in future its application in finding optimized combination of Chinese herbal mixture.

## Supplementary Material

Fig. 1S A typical DE/rand/1/bin strategy introduced in optimization of flavonoid combinations [21]. Initially, NP (number of population) combinations of the four flavonoids were generated. For each original combination xi, a mutation combination vi was generated following the formula in step 2, where r1, r2 and r3 are randomly generated integers in the range of 1~NP. Then the originals xi (black border) and mutations vi (orange border) were crossed to produce crossover combinations ui, controlled by the crossover constant CR in step 3. The crossover combinations were used as the trial combinations to compete with the original combinations. Combinations that derived better desired biological activities were carried over to the new generation xi,new. We decided to start with NP=6 and CR=0.5 to achieve fast convergence, and gradually increase to NP=18 and CR=0.9 to avoid being trapped by localized maximum responses. The brightness of the colors represented different concentration levels of each flavonoid
Fig. 2S The median effect equation for computerized simulation of synergism, additivism and antagonism of the effect of multiple drugs: in optimization of flavonoid combinations, the fraction affected fa was expressed as fa=C/T, where C was the control response, and T represented drug induced response [26].
Fig. 3S Benchmark test results of four functions for 50 times: (A) number of trials required to find the solutions of the four functions using DE algorithm and the exhaustive algorithm (Rand). Differential evolution (DE) solved the problems within only 58 ± 11, 53 ± 11, 131 ± 103 and 90 ± 46 trials for the four functions; (B) minimum value found by both algorithms in 60 trials; With DE, the minimum values were 0.42 ± 0.61, 0.38 ± 0.57, 25.48 ± 43.38, and 0.31 ± 0.23, and out of the 50 runs, there were 32, 33, 22, and 11 runs, that the minimum value 0 was found in 60 trials for the four functions, respectively.
