# Supplementary material for: Optimizing Combinations of Flavonoids Deriving from Astragali Radix in Activating the Regulatory Element of Erythropoietin by a Feedback System Control Scheme
Source: Evid Based Complement Alternat Med. 2013 Apr 30;2013:541436. doi: 10.1155/2013/541436 (PMC3657416; doi:10.1155/2013/541436)
Supplement: Supplementary file 1 — Fig. 1S A typical DE/rand/1/bin strategy introduced in optimization of flavonoid combinations [21]. Initially, NP (number of population) combinations of the four flavonoids were generated. For each original combination xi, a mutation combination vi was generated following the formula in step 2, where r1, r2 and r3 are randomly generated integers in the range of 1~NP. Then the originals xi (black border) and mutations vi (orange border) were crossed to produce crossover combinations ui, controlled by the crossover constant CR in step 3. The crossover combinations were used as the trial combinations to compete with the original combinations. Combinations that derived better desired biological activities were carried over to the new generation xi,new. We decided to start with NP=6 and CR=0.5 to achieve fast convergence, and gradually increase to NP=18 and CR=0.9 to avoid being trapped by localized maximum responses. The brightness of the colors represented different concentration levels of each flavonoid Fig. 2S The median effect equation for computerized simulation of synergism, additivism and antagonism of the effect of multiple drugs: in optimization of flavonoid combinations, the fraction affected fa was expressed as fa=C/T, where C was the control response, and T represented drug induced response [26]. Fig. 3S Benchmark test results of four functions for 50 times: (A) number of trials required to find the solutions of the four functions using DE algorithm and the exhaustive algorithm (Rand). Differential evolution (DE) solved the problems within only 58 ± 11, 53 ± 11, 131 ± 103 and 90 ± 46 trials for the four functions; (B) minimum value found by both algorithms in 60 trials; With DE, the minimum values were 0.42 ± 0.61, 0.38 ± 0.57, 25.48 ± 43.38, and 0.31 ± 0.23, and out of the 50 runs, there were 32, 33, 22, and 11 runs, that the minimum value 0 was found in 60 trials for the four functions, respectively. [file 541436.f1.pdf]

Supporting information

Optimizing Combinations of Flavonoids Deriving from Astragali Radix in Activating the Regulatory Element of Erythropoietin by a Feedback System Control Scheme

Hui Yu,[1a] Wendy L. Zhang,[2a] Xianting Ding,[3] Ken Y. Z. Zheng,[2] Chih-Ming Ho,[3] Karl W. K. Tsim,[2*] and Yi-Kuen Lee[1*]

[1] *Department of Mechanical Engineering, The Hong Kong University of Science and Technology, Clear Water Bay Road, Hong Kong, China*

[2] *Division of Life Science and Center for Chinese Medicine, The Hong Kong University of Science and Technology, Clear Water Bay Road, Hong Kong, China*

[3] *Center for Cell Control, Mechanical and Aerospace Engineering Department, Biomedical Engineering Department, University of California, Los Angeles, California 90095-1597, USA*

*Correspondence should be addressed to:

Dr. Yi-Kuen Lee, Department of Mechanical Engineering, The Hong Kong University of Science and Technology, Clear Water Bay Road, Hong Kong; Tel: (852) 2358 8663; Fax: (852) 2358 1543; E-mail: meyklee@ust.hk; OR

Dr. Karl Tsim, Center for Chinese Medicine, The Hong Kong University of Science and Technology, Clear Water Bay Road, Hong Kong; Tel: (852) 2358 7332; Fax: (852) 2358 1559; E-mail: botsim@ust.hk

[a] These authors contributed equally to this work.

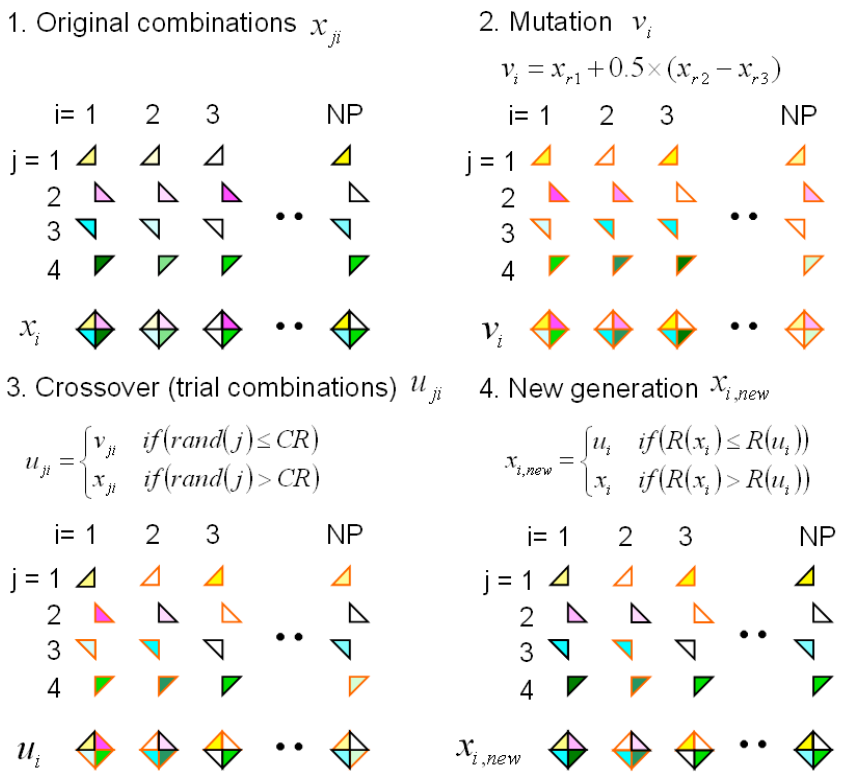

Fig. 1S A typical DE/rand/1/bin strategy introduced in optimization of flavonoid combinations [21]. Initially, NP (number of population) combinations of the four flavonoids were generated. For each original combination x_i, a mutation combination v_i was generated following the formula in step 2, where $r1$, $r2$ and $r3$ are randomly generated integers in the range of 1~NP. Then the originals x_i (black border) and mutations v_i (orange border) were crossed to produce crossover combinations u_i, controlled by the crossover constant CR in step 3. The crossover combinations were used as the trial combinations to compete with the original combinations. Combinations that derived better desired biological activities were carried over to the new generation $x_{i,new}$. We decided to start with NP=6 and CR=0.5 to achieve fast convergence, and gradually increase to NP=18 and CR=0.9 to avoid being trapped by localized maximum responses. The brightness of the colors represented different concentration levels of each flavonoid.

Fig. 1S

Yu et al 2013

The Median Effect Equation

$$(1)\ f_a/f_u = (D/D_m)^m$$

$$(2)\ Log(f_a/f_u) = mlog(D) - mlog(D_m)$$

$$(3)\ f_a = 1/[1 + (D_{m/D})^m]$$

$$(4)\ D_x = D_m[f_a/(1 - f_a)]^{1/m}$$

D = Dose

f_a = fraction affected

f_u = fraction unaffected

D_m = median-effect dose

m = slope, or kinetic order

$$(5)\ CI = \frac{(D)_1}{(D_x)_1} + \frac{(D)_2}{(D_x)_2} = \frac{1}{(DRI)_1} + \frac{1}{(DRI)_2}$$

$$\begin{bmatrix} (D_x)_{1,2} = (D)_1 + (D)_2 \\ and\ (D)_1/(D)_2 = P/Q \end{bmatrix}$$

$$(D)_1 = (D_x)_{1,2} \times P/(P + Q)$$

$$(D)_2 = (D_x)_{1,2} \times Q/(P + Q)$$

CI = Combination index

CI = 1 (Summation)

< 1 (Synergism)

> 1 (antagonism)

DRI = Dose-Reduction Index

$$(DRI)_1 = \frac{(D_x)_1}{(D)_1}, (DRI)_2 = \frac{(D_x)_2}{(D)_2}$$

For n Drug Combinations:

$$CI = \sum_{j=1}^{n} \frac{(D)_j}{(D_x)_j}$$

Fig. 2S The median effect equation for computerized simulation of synergism, additivism and antagonism of the effect of multiple drugs: in optimization of flavonoid combinations, the fraction affected f_a was expressed as $f_a=C/T$, where C was the control response, and T represented drug induced response [26].

Fig. 2S

Yu et al 2013

Table. 1S Definition of the four benchmark functions

Name	Definition (n=4)	Search space
Sphere	$f(x) = \sum_{i=1}^{n} x_i^2$	$[-2, -1, 0, 1, 2, 3]^4$
Rastrigin	$f(x) = 10n + \sum_{i=1}^{n} \left(x_i^2 - 10\cos(2\pi x_i)\right)$	$[-2, -1, 0, 1, 2, 3]^4$
Rosenbrock	$f(x) = \sum_{i=1}^{n-1} \left[100\left(x_i^2 - x_{i+1}\right)^2 + \left(x_i - 1\right)^2\right]$	$[-2, -1, 0, 1, 2, 3]^4$
Griewank	$f(x) = \sum_{i=1}^{n-1} \frac{x_i^2}{4000} - \prod_{i=1}^{n} \cos\left(x_i/\sqrt{i}\right) + 1$	$[-20, -10, 0, 10, 20, 30]^4$

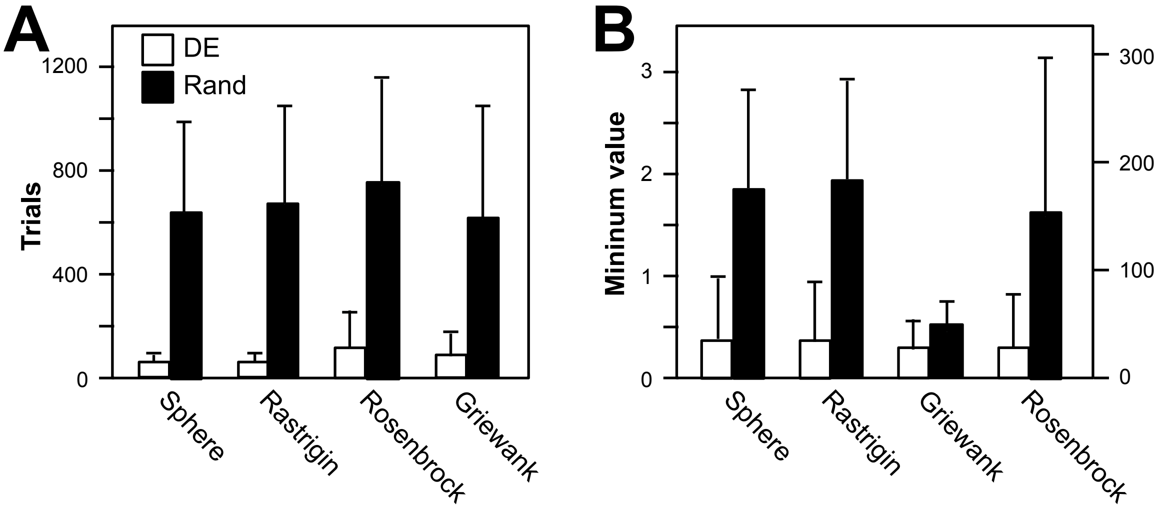

Fig. 3S Benchmark test results of four functions for 50 times: (**A**) number of trials required to find the solutions of the four functions using DE algorithm and the exhaustive algorithm (Rand). Differential evolution (DE) solved the problems within only 58 ± 11, 53 ± 11, 131 ± 103 and 90 ± 46 trials for the four functions; (**B**) minimum value found by both algorithms in 60 trials; With DE, the minimum values were 0.42 ± 0.61, 0.38 ± 0.57, 25.48 ± 43.38, and 0.31 ± 0.23, and out of the 50 runs, there were 32, 33, 22, and 11 runs, that the minimum value 0 was found in 60 trials for the four functions, respectively.

Fig. 3S

Yu et al 2013
